# Design, Development and Construct Validation of the Children’s Dietary Inflammatory Index

**DOI:** 10.3390/nu10080993

**Published:** 2018-07-30

**Authors:** Samira Khan, Michael D. Wirth, Andrew Ortaglia, Christian R. Alvarado, Nitin Shivappa, Thomas G. Hurley, James R. Hebert

**Affiliations:** 1 Statewide Cancer Prevention and Control Program (CPCP), Arnold School of Public Health, University of South Carolina, 915 Greene Street, Columbia, SC 29208, USA; khans@mailbox.sc.edu (S.K.); wirthm@mailbox.sc.edu (M.D.W.); alvarac@email.sc.edu (C.R.A.); shivappa@mailbox.sc.edu (N.S.); thurley@mailbox.sc.edu (T.G.H.); 2 Connecting Health Innovations, LLC, Columbia, SC 29201, USA; skhan@chi-llc.net (S.K.); mwirth@chi-llc.net (M.D.W.); nshivappa@chi-llc.net (N.S.); thurley@chi-llc.net (T.G.H.); 3 Epidemiology and Biostatistics, Arnold School of Public Health, University of South Carolina, 915 Greene Street, Columbia, SC 29208, USA; ORTAGLIA@mailbox.sc.edu; 4 College of Nursing, University of South Carolina, Columbia, SC 29208, USA

**Keywords:** diet, inflammation, children’s-dietary inflammatory index

## Abstract

Objective: To design and validate a literature-derived, population-based Children’s Dietary Inflammatory Index (C-DII)^TM^. Design: The C-DII was developed based on a review of literature through 2010. Dietary data obtained from children in 16 different countries were used to create a reference database for computing C-DII scores based on consumption of macronutrients, vitamins, minerals, and whole foods. Construct validation was performed using quantile regression to assess the association between C-reactive protein (CRP) concentrations and C-DII scores. Data Sources: All data used for construct validation were obtained from children between six and 14 years of age (*n* = 3300) who participated in the U.S. National Health and Nutrition Examination Survey (NHANES) (2005–2010). Results: The C-DII was successfully validated with blood CRP concentrations in this heterogeneous sample of 3300 children from NHANES (52% male; 29% African American, 25% Mexican American; mean age 11 years). The final model was adjusted for sex, age, race, asthma, body mass index (BMI), and infections. Children in level 3 (i.e., quartiles 3 and 4 combined) of the C-DII (i.e., children with the most pro-inflammatory diets) had a CRP value 0.097 mg/dL higher than that in level 1 (i.e., quartile 1) for CRP values at the 75th percentile of CRP using quantile regression (*p* < 0.05). Conclusion: The C-DII predicted blood CRP concentrations among children 6–14 years in the NHANES. Further construct validation with CRP and other inflammatory markers is required to deepen understanding of the relationship between the C-DII and markers of inflammation in children.

## 1. Introduction

Inflammation is regulated in the body through a variety of processes that involve intercellular signaling via well-characterized cytokine and chemokine messengers [1,2,3,4]. An acute inflammatory response is necessary for normal physiologic functioning, including response to infectious disease agents [5,6,7,8,9]. This acute inflammatory response is characterized by an increase in vascular permeability and blood flow accompanied by the accumulation of inflammatory mediators, fluid, and leukocytes. Acute inflammatory responses are time-limited and require negative feedback signaling between pro-inflammatory cytokines that turn on the response and anti-inflammatory cytokines that signal acute inflammatory responses to cease [10,11]. Because of the unique nutritional needs of children, and because it is imperative to deal with enteric and other infections, to which children are especially vulnerable, much of the initial focus of the field of immunology was on children and childhood nutrition [9,12].

In contrast to acute inflammation, chronic systematic inflammation results when negative feedback does not occur (or is either incomplete or inefficient) [10,13]. The chronic phase is characterized by a specific cellular immune response along with specific humoral responses [14,15]. In addition to its role in regulating acute inflammatory responses, diet also has been implicated in regulating chronic inflammation [16,17].

The Dietary Inflammatory Index (DII^®^) was developed to classify human dietary patterns on a continuous scale from anti-inflammatory to pro-inflammatory [18]. Although the original DII could predict changes in C-reactive protein (CRP) levels, a newer version of the DII was developed that reflected an update in the review of peer-reviewed articles by adding those published from 2007 to 2010 and a refined scoring algorithm [19]. Subsequently, the new DII has been construct validated in nine studies against inflammatory biomarkers in different populations and under varying conditions [20,21,22,23,24,25,26].

In the process of publishing the new DII, we observed that the relationship between total caloric intake and DII score is highly idiosyncratic across populations, and by body size. For example, in most populations, DII scores decrease with increasing caloric intake [27,28,29]. This observation led, in part, to development of the Energy Density-DII (E-DII) and then to the Children’s Dietary Inflammatory Index (C-DII^TM^). In addition, the world standard database used for the DII includes data on dietary parameters only from adults, and not all the parameters that comprise the DII (e.g., alcohol intake) are appropriate to include when evaluating children’s dietary intake. With these limitations in mind, we set out to create a “world” standard database of food parameters that could be used to calculate C-DII scores among children. At the same time, it was understood that construct validation of the C-DII was necessary. Therefore, we also sought to conduct a construct validation to test the relationship between C-DII scores and levels of an inflammatory biomarker, CRP. We hypothesized that children with more pro-inflammatory diets (i.e., higher C-DII scores) will have higher values of CRP compared to children with lower C-DII scores.

This paper describes the development of the C-DII and the relationship between C-DII scores and blood concentrations of CRP in a nationally representative sample of children. 

## 2. Methods

### 2.1. C-DII^TM^ Development

To develop the C-DII, methods similar to those used to develop the current version of the DII were employed [19]. First, the “inflammatory effect scores”, which were derived from an extensive literature search to develop the DII, were still applicable and therefore used for the development of the C-DII. The Dietary Inflammatory Index (DII^®^) was developed to classify human dietary patterns on a continuous scale from anti-inflammatory to pro-inflammatory [18]. Although the original DII could predict changes in CRP levels, a newer version of the DII was developed that reflected an update in the review of peer-reviewed articles by adding those published from 2007 to 2010 and a refined scoring algorithm [19]. Subsequently, the new DII has been construct validated in nine studies against inflammatory biomarkers in different populations and under varying conditions [20,21,22,23,24,25,26]. This work entailed a literature review of 1943 articles that was conducted to identify food parameters which are associated with six inflammatory biomarkers: Interleukin-1beta (IL-1β), interleukin-4 (IL-4), interleukin-6 (IL-6), interleukin-10 (IL-10), tumor necrosis factor-alpha (TNF-α), and CRP. By contrast with the constrained list of the six inflammatory biomarkers, the list of dietary parameters was open. For each article reviewed, a score was assigned for each food parameter based on its effect on inflammation. A ‘+1’ was assigned if the effect of the parameter was pro-inflammatory, a ‘−1’ was assigned if the effect of the parameter was anti-inflammatory; and ‘0’ was assigned if the effect of the parameter was neutral. Each food parameter for which a finding existed was assigned a score for each article separately [19]. Published articles were weighted by study design, with highest weight assigned to experimental studies in humans and the lowest weight assigned to cell culture experiments. Based on the weights, pro- or anti-inflammatory fractions were calculated for each food parameter. Next, the overall inflammatory effect score specific to each food parameter was calculated by subtracting the anti-inflammatory fraction from pro-inflammatory fraction [19].

The food parameters used to calculate C-DII scores are: vitamin A, thiamine, riboflavin, niacin, vitamin B6, folic acid, vitamin B12, vitamin D, vitamin C, vitamin E, beta carotene, energy, carbohydrates, fiber, total fat, saturated fat, mono-unsaturated fatty acid (MUFA), poly-unsaturated fatty acid (PUFA), cholesterol, protein, alcohol, iron (Fe), magnesium (Mg), selenium (Se), and zinc (Zn).

#### Developing a Composite Database Representing a Diversity of Children’s Diets

Dietary intakes from a wide range of diverse populations from different countries representing six continents were used to construct a composite database for the C-DII. The methodology used was virtually identical to that employed for developing the DII for adults. Data collection began in August 2016 and ended in May 2017. Using the National Library of Medicine database (Medline), we identified 35 different published papers with sample size over 200 with children’s diet data collected using either a food frequency questionnaire (FFQ) or diet recalls. Overall, data were collected from 16 different countries representing diverse diets from six different continents.

Dietary data for creating a global database were collected from three different sources: (1) datasets (*n* = 11) received directly from the study principal investigators (from collaborations during DII development); (2) from published articles (*n* = 3); (3) National Health and Nutrition Examination (NHANES) Survey reports (*n* = 2). We sent emails to the authors of the 35 published articles with sample size >200 to obtain consent to use data. After three attempts to obtain consent to use data, we finalized a list of the articles from which we could extract dietary data for the global database. The decision was based on the availability of macro and micronutrients presented in the tables in the articles for children age 6–14 years.

The world database used for the C-DII contains dietary information from the following countries (and sources): (i) USA—the NHANES data set 2005–2011 [30]; (ii) Australia—mean values were taken from the National Nutrition Survey report of 1999 [31]; (iii) Japan—means were taken from the National Nutrition Survey Report [32]; (iv) Korea—mean values were taken from the Korean National Health and Nutrition Examination Survey (KNHANES); (v) Spain, (vi) Belgium, (vii) Greece, (viii) Germany, (ix) France, (x) Italy, (xi) Sweden, and (xii) Austria—means were taken from the Healthy Lifestyle in Europe by Nutrition in Adolescence (HELENA) study [33]; (xiii) Venezuela—means were taken from an article published by Bernal et al. [34]; (xiv); United Arab Emirates—means were taken from an article published by Ali et al. [35]; and (xvi) Chile—means were taken from an article published by Liberona et al. [36].

Missing food parameters for countries included in the database were left blank, and the overall mean and standard deviation were calculated using only data from the datasets that had information on that specific food parameter. For example, the mean and standard deviation for vitamin A were calculated using 13 countries because South Africa, Venezuela, and Chile did not have information on that food parameter. Some sources of data provided mean intake values separately for males and females or for different age groups within the range of 6–14 years; in such cases, the values were averaged. For example, the data from the United Emirates, gathered from the article by Ali et al. [35], had children separated by sex, and by age group: 6–8, 9–13, and 14–18 years. In this case, the values were averaged first between age groups for each sex, and then averaged between males and females. The list of 25 food parameters and the information regarding each country (present or missing for each food parameter) is presented in Supplementary Table S1. 

Individual estimates of consumption were standardized to a global database of children’s dietary intake, in a manner analogous to methods used to compute adult DII scores. As children’s diet differs from the adult diet, one difference between the DII and the C-DII is that the C-DII identified only 25 food parameters, compared to the 45 used in scoring of the adult DII.

### 2.2. Calculation of the Children’s Dietary Inflammatory Index

Calculation of the C-DII is based on the dietary intake data that are related to the regionally representative world database. These values become multipliers to express an individual child’s exposure relative to the ‘standard global mean’ as a Z-score. This is done by subtracting the ‘standard mean’ from the reported amount and dividing this value by the global standard deviation. This Z-score is then converted to a proportion to avoid ‘right skewing’. A symmetrical distribution was achieved with values centered on 0 (null) and bounded between −1 (maximally anti-inflammatory) and +1 (maximally pro-inflammatory), by doubling each proportion and then subtracting ‘1’. This centered proportion score is then multiplied by the ‘overall food parameter-specific inflammatory effect score’. In the final step, ‘food parameter-specific DII scores’ are added to create the ‘overall C-DII score’ for an individual child. This approach eliminates the problem of non-comparability of units because the Z-scores and centered proportion scores are independent of the units of measurement [19]. This technique is the same as that used previously in the development of the DII [19]. Additionally, the C-DII, just like recent developments in the DII, can be calculated per 1000 calories consumed to take into account differing amounts of energy consumption between people. 

### 2.3. Validation Study: NHANES Study Population

The NHANES survey examines a nationally representative sample of about 10,000 persons each year, and it employees a complex, multistage probability sampling design and generates weights to create a nationally representative dataset of the US population. More detailed information about the NHANES methods and protocols can be found on the Centers for Disease Control and Prevention (CDC)—National Center for Health Statistics website [37].

The proposed study population was restricted to children age 6–14 years (primary inclusion criterion) from the NHANES dataset (2005–2010) who had complete dietary data, demographics, and blood results, including CRP. There were 3445 children with CRP information and 145 were removed due to missing information. The final sample for analysis included data from 3300 children.

### 2.4. Dietary Assessment

NHANES 24-h dietary recall data were used to calculate C-DII scores. These data were collected through in-person interviews conducted by trained dietary interviewers fluent in English and Spanish. These staff members and the USDA’s Food Survey Research Group were responsible for the dietary data collection, maintenance of the databases, and data processing. The food parameters from the NHANES data set included carbohydrates, protein, fat, fiber, fatty acids, vitamins (A, B1, B2, B6, B12, C, D, E), iron, magnesium, zinc, selenium, folic acid and beta carotene. To account for total energy intake, the C-DII was calculated per 1000 calories of food consumed.

### 2.5. CRP Data 

The blood samples for CRP determination were processed, stored and analyzed at the University of Washington, Seattle, WA, using Behring Nephelometer for quantitative CRP determination (NHANES 2009–2010 data documentation, 2011). The blood specimens for children aged 3 years and older were collected in a mobile examination clinic by using regular or serum-separator vacutainers, and specimens were kept frozen at <−20 °C if testing was not done within 24 h of specimen collection. The serum or plasma was separated from the cells within 60 min of collection; recommended sample volume for assay is 1.0 mL. Specimens are stored in glass or plastic vials and kept tightly sealed. The lower detection limit for CRP was 0.02 ng/mL.

### 2.6. Study Population and Covariates

Study covariates included demographic characteristics, such as age, sex, race, body mass index (BMI kg/m^2^), ethnicity, self-reported asthma, and infection at the time of data collection. Subjects with missing information on any of these variables were removed from the analysis. 

### 2.7. Statistical Analyses

The statistical software SAS 9.4^®^ (SAS Institute, Cary, NC, USA) and R 3.4.3^®^ (The R Foundation, Vienna, Austria) were used for analyses. For descriptive analysis, we report continuous variables with means and standard deviation (SD) and categorical variables using frequencies and percentage across C-DII quartiles. Chi Square tests were performed for descriptive statistics of categorical covariates across C-DII quartiles, and Analysis of Variance (ANOVAs) were used for the continuous covariates (See Table 1 for a list of covariates). 

The C-DII scores were expressed as quartiles, and CRP weighted percentiles were used for analysis (see Table 2 for percentile details). For this analysis, we combined quartiles 3 and 4 of the C-DII because the ranges in values for these quartiles were somewhat narrow and, together, represented the most pro-inflammatory diets. Also, combining these two quartiles provided a larger sample size for the comparison of interest, therefore, providing more statistical power. For these analyses, C-DII scores were expressed as three levels, with 1 = quartile 1 (anti-inflammatory); 2 = quartile 2 (neutral); and 3 = quartiles 3 + 4 (most pro-inflammatory). For all comparative analyses, C-DII quartile 1 was used as the reference. Categorizing the C-DII initially into quartiles was done because the DII is typically analyzed using quartiles, and C-DII follows strategies and methodologies used to assess and validate the DII [22,24].

Quantile regression was used to assess the association between C-DII levels and weighted CRP, treated as the dependent variable, across adjusted CRP percentiles (25th, 50th, 75th and 90th) after controlling for sex, age, race, asthma, BMI, and infections. “R” version 3.43 was used for the quantile regression analysis, with the survey package extended to accommodate quantile regression, accounting for the NHANES complex survey design using standard errors calculated via replicate weights [38].

**Figure 1:**
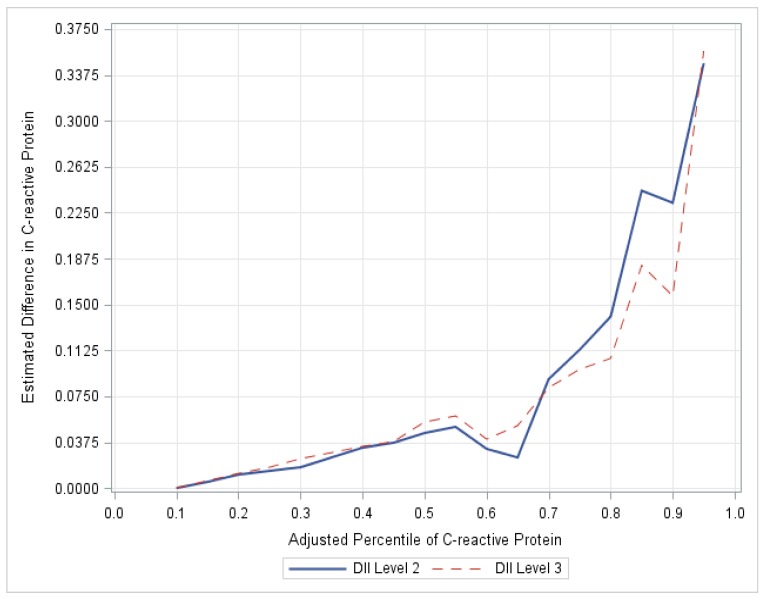
Plot of regression coefficients (C-DII and CRP). The magnitude of the differences in CRP increases at the upper percentiles up to the 85th percentile. CRP for children in C-DII group 2 as compared to C-DII group 1 at the 85th percentile is over 5 times the difference in CRP at the median, NHANES, 2005–2012.To reinforce the validity of our study, a logistic regression was performed using dichotomized CRP as the dependent variable (designated as high risk if CRP ≥ 0.50 mg/L or low risk <0.50 mg/dL) using a median cut-point. The odds of a CRP value ≥ 0.50 mg/L were obtained for those in C-DII level 3 compared to level 1, controlling for sex, age, race, asthma, BMI, and infections.

Quantile regression has the advantage of allowing the examination of relationships across the entire CRP distribution, allowing for a comprehensive evaluation of the association between C-DII and CRP. Quantile regression coefficients are interpreted similarly to mean regression coefficients except that a quantile regression coefficient indicates the change in the value at the modeled percentile, not the mean, of the dependent variable. For example, consider a categorical predictor such as C-DII classified by quartile, with the lowest C-DII quartile being the reference level. A coefficient estimate of 0.1 mg/L for the second quartile of C-DII in the quantile regression model for the 90th percentile would indicate that the 90th percentile of CRP is estimated to be 0.1 mg/L greater for children in the second C-DII quartile as compared to children in the first C-DII quartile after controlling for other covariates in the model (See Figure 1 for a depiction).

## 3. Results

Characteristics of children from the NHANES database (2005–2010 two-year cycles) whose data were used to validate C-DII scores are presented in Table 1. Weighted mean CRP value (*n* = 3193) was 0.84 mg/dL (standard error (SE) = 0.03). C-DII mean score was +0.99 (SE = 0.05), with values ranging from a maximally anti-inflammatory value of −3.98 to a maximally pro-inflammatory value of +4.39. Participants in quartile 1 had the least inflammatory scores (−3.98 to −0.04) and children in upper quartiles 3 and 4 had the most pro-inflammatory diet (2.07 to 4.39). The majority (≈82%) of the children in all quartiles were normal weight. More children in upper quartiles 3 and 4 reported asthma (17.4% and 19.7%, respectively) (*p* < 0.01) compared to lower quartiles (15.1% and 16.1%). 

Significant differences in CRP were observed at the adjusted 50th and 75th percentiles for both groups, that is levels 2 (quartile 2) and 3 (quartiles 3 + 4 combined). Differences in CRP for children in C-DII level 2 as compared to C-DII level 1 were 0.045 and 0.113 mg/dL at the 50th and 75th percentiles, respectively. Similarly, differences in CRP for children in C-DII level 3 as compared to C-DII level 1 were 0.054 and 0.097 mg/dL at the 50th and 75th percentiles, respectively (see Table 2). The estimates for the association of C-DII on the adjusted 25th, 50th, 75th and 90th percentile CRP values are shown in Table 2. 

For both C-DII levels 2 and 3, the magnitude of the estimated impact of C-DII tended to increase with increasing CRP percentiles. The magnitude of the differences in CRP between C-DII level 3 and 1 increase up to the 85th percentile. The estimated difference in CRP for children in C-DII level 2, as compared to C-DII level 3, at the 85th percentile is over 5 times the difference in CRP at the median (see Figure 1). The regression coefficients at the 90th percentile did not reach the nominal cut-point for statistical significance because the standard errors also increased, which tends to occur at the upper tail of the distribution.

Based on the logistic regression results, children in C-DII levels 2 and 3 had increased odds of having a CRP value above 0.50 mg/L. Specifically, children in C-DII level 2 (which equates to quartile 2) had 22% increased odds (95% confidence intervals (CI) = 0.94–1.59) of a CRP value above 0.50 mg/L compared to children in C-DII level 1. However, this did not achieve statistical significance. Children in C-DII level 3 (which equates to C-DII quartiles 3 and 4) had statistically significant 38% increased odds of an elevated CRP compared to children in C-DII level 1 (odds ratio (OR) = 1.38, 95% CI = 1.11–1.71, data not tabulated). 

We also conducted sensitivity analyses, limiting the data set to observations for children without asthma and without an infection (as suggested by the reviewer) (Supplementary Table S2). This deletion of 1239 observations, or about 40% of the previous unweighted sample size, reduced the sample size from 3112 to 1873. There was no change in the direction of any regression coefficient. The only noteworthy difference between analyses is that the coefficient for the 90th percentile for CDII quartile 3 became statistically significant. Due to the significant reduction in the total number of observations, the appropriateness of the NHANES supplied survey weights becomes questionable. 

## 4. Discussion

Results from this study showed that: (1) We were able to construct a global reference database for C-DII analogous to what we had done for the DII; and (2) Results from the construct validation indicated that the resulting C-DII scores were associated with CRP values among children 6 to 14 years of age in the NHANES data set. Analyses were performed to examine the association between three levels of the C-DII and the complete distribution of CRP using quantile regression. The major finding from these analyses is that CRP levels differ across the levels of the C-DII and the association of the C-DII is not constant across the CRP distribution. Specifically, we found that in level 2 of the C-DII there was significant increase in levels of CRP at the 50th, 55th, 70th, 75th, 80th, and 85th percentiles. This also is consistent with previous studies that have shown that quantile regression allows for the examination of outcomes at multiple levels, and is not influenced by outliers or skewness of the dependent variable [39]. Results in level 3 of the C-DII, representing the 3rd and 4th C-DII quartiles were more strongly in support of our hypothesis. Increased levels of the C-DII are associated with an increase in levels of CRP which is a risk factor for obesity and chronic disease.

Obesity is a pro-inflammatory state and childhood obesity is strongly associated with chronic inflammation and is on the rise [40,41,42]. It is known that obese children often become obese adults [43] and are more likely to suffer from insulin resistance [44], high blood pressure, unhealthy levels of serum lipids, and increased cardiovascular disease (CVD) risk [45]. This obesity epidemic is the result of specific changes in our environment and health-related behaviors [46,47]. High-calorie, inexpensive foods have become widely available and are heavily advertised [47,48]. Therefore, it is important to develop tools that can help monitor children’s diet to tackle childhood obesity and its important correlate, inflammation.

Diet plays an essential role in the regulation of chronic inflammation [18,49,50,51]. Western diets rich in red meat, high-fat, sugar, dairy products, and refined grains have been associated with higher levels of CRP and other inflammatory markers [52,53]. Several studies have investigated individual food items such as meat or individual nutrients, such as vitamin C, and have observed associations with inflammation [54,55,56,57,58,59]. However, it is important to note that nutrients and individual foods are rarely consumed alone in a diet. Therefore, a nutrient or individual food effect may not be independent of the effect of other nutrients in the diet. Until recently, no dietary index was developed to predict inflammatory potential of whole diet; i.e., not just individual items or nutrients. Few research studies have investigated the effects of dietary indices such as the Healthy Eating Index (HEI), and Mediterranean Diet scores [60,61]. Therefore, there was a need to develop a comprehensive tool that can help children and their parents make healthier food choices that, in turn, can help reduce the risk of chronic diseases by directly influencing inflammation and preventing, or at least greatly limiting, weight gain.

The DII was developed and validated against inflammatory markers in adult populations and has been associated with a range of outcomes including cancer, metabolic syndrome, and asthma [19,62,63,64,65]. An important component of the DII calculation is the standardization of food parameter intake estimates to a “global” database of intake for the food parameters [19]. Furthermore, in developing the DII we identified eleven food consumption data sets from different parts of the world to represent a range of dietary intakes, which was used as the ‘reference’ database [19]. However, DII use among younger individuals (e.g., 6–14 year olds) is limited because of the “global” database used for this index. There are several barriers that prevent use of the current DII among children. The “global” standard database includes only adult dietary intakes and not all of the food parameters that comprise the DII (e.g., alcohol) may be appropriate for children dietary intake. Furthermore, we could not obtain data on garlic from any dataset we collected for the global database. Therefore, we developed C-DII, which can provide guidance for improving diets among children, and in combating obesity. Childhood obesity has been on the rise in the US, and obese children are more likely to become obese adults who experience risk factors associated with chronic disease risk [40,41,42,43,45]. Therefore, it is imperative that we create tools to monitor dietary patterns that can contribute to fluctuations in inflammation levels in the body resulting in obesity and chronic diseases [50,66]. To address this need, we developed the C-DII, which is based on the scoring algorithm of the DII. 

A necessary step in the process was to identify dietary data collected in children from around the world. A total of 16 such data sets were obtained that included the US NHANES [30], the Korean NHANES [67], HELENA study (in ten European cities in Greece, Germany, Belgium, France, Hungary, Italy, Sweden, Austria, and Spain) [33], and a dataset from South Africa [68] that helped create the “world” database for C-DII. 

The C-DII represents an innovative tool for evaluating the inflammatory potential of children’s diet and it can be applied to any child population in which dietary information of sufficient quality has been gathered. The C-DII is not limited to data from 24-h recalls but can be used with a wide range of dietary data from various sources, including children-appropriate food frequency questionnaires (FFQ). The C-DII can be used to establish intervention strategies regarding an individual child’s dietary goals to reduce inflammation and the risk of various health conditions [19].

This study has several strengths. It is the first study to develop and validate an index to explore the inflammatory potential of children’s diet based on a global database. Second, it provided a unique opportunity to assess the inflammatory potential of diet in relation to CRP in a nationally representative sample. Third, it was possible to control for several confounding factors such as age, sex, race, BMI, and infection, and asthma.

Despite its strengths, there were several limitations to this study including a relative paucity of robust and reliable data on child nutrition. Regular public health surveillance of child nutrition occurs infrequently around the world, and few research studies have collected children dietary data, so to find reliable sources for these data was challenging, and not all datasets provided 100% complete dietary data. Additionally, we were limited by the availability of dietary data (i.e., a single 24-h dietary recall) in the NHANES. 

## 5. Conclusions

Results indicate that the C-DII predicts CRP among children aged 6 to 14 years, and it appears that the C-DII is more strongly predictive of CRP among the higher levels CRP compared to lower levels of CRP. The C-DII can be used as a means for informing primary prevention and for educating physicians and other providers, parents and children on the importance of a healthy diet to reduce chronic disease rates and to enhance feelings of well-being and to improve quality of life. Further construct validation is required to deepen the understanding of the relationship between the C-DII and markers of inflammation in children. Additionally, more research should be focused on the role of diet in inflammation-related conditions in children.

## Figures and Tables

**Table 1:** Participants’ characteristics across quartiles of the children’s dietary inflammatory index (C-DII) among 3300 children. NHANES, 2005–2012.

		C-DII Quartiles			
	1st	2nd	3rd	4th	
Participant	(−3.99, −0.04)	(−0.05, 1.14)	(1.14, 2.07)	(2.08, 4.39)	*p*-value ^a^
Characteristics					
Age (years)					<0.0001
	10.0 ± 3.0	11.0 ± 3.0	11.0 ± 3.0	11.0 ± 3.0	
Sex					0.29
Male	411 (48.52)	430 (51.62)	413 (49.64)	409 (51.9)	
Female	436 (51.48)	403 (48.38)	419 (50.36)	379 (48.1)	
Race					<0.0001
Non-Hispanic Black	180 (21.25)	212 (25.45)	223 (26.8)	229 (29.06)	
Non-Hispanic White	224 (26.45)	228 (27.37)	229 (27.52)	251 (31.85)	
Mexican American	323 (38.13)	267 (32.05)	262 (31.49)	198 (25.13)	
Other	120 (14.17)	126 (15.13)	118 (14.18)	110 (13.96)	
Body mass index (tertiles) ^b,c,d^					0.19
I	705 (83.43)	682 (82.17)	671 (81.04)	640 (81.42)	
II	82 (9.7)	103 (12.41)	101 (12.2)	84 (10.69)	
III	58 (6.69)	45 (5.42)	56 (6.76)	62 (7.89)	
Asthma ^e^					0.01
Yes	128 (15.11)	134 (16.13)	145 (17.45)	155 (19.7)	
No	719 (84.89)	697 (83.87)	686 (82.55)	632 (80.3)	
Infection ^e^					0.06
Yes	252 (27.39)	237 (25.76)	225 (24.46)	206 (22.39)	
No	571 (24.72)	579 (25.06)	595 (25.76)	565 (24.46)	

^a^*p* Value for Chi-square test; ^b^ BMI (kg/m^2^): Category I Under or Normal weight (i.e., <25 kg/m^2^); ^c^ BMI (kg/m^2^): Category II Overweight (i.e., ≥25 kg/m^2^ and <30 kg/m^2^); ^d^ BMI (kg/m^2^): Category III Obese (i.e., ≥30 kg/m^2^); ^e^ Asthma and Infection are self-reported; Column percentages may not equal 100% due to rounding. Stratum total may not equal column totals due to missing data. For categorical covariates, frequencies (%) were presented, and chi-square tests were used to derive *p*-values. For continuous covariates, means and standard deviations were presented, and ANOVAs were used to derive *p*-values.

**Table 2:** The quantile regression coefficients for children’s dietary inflammatory index treated categorically with 3 levels (level 1 is the reference level) adjusted for sex, age, race, asthma, BMI and infection, NHANES, 2005–2012 *.

Weighted CRP Levels												
	25th Percentile			50th Percentile			75th Percentile			90th Percentile		
	Est.	SE	95% CI	Est.	SE	95% CI	Est.	SE	95% CI	Est.	SE	95% CI
C-DII Level												
2 (Quartile 2)	0.014	0.009	(−0.004, 0.032)	† 0.045	0.020	(0.005, 0.085)	† 0.113	0.055	(0.005, 0.221)	0.233	0.192	(−0.144, 0.609)
3 (Quartiles 3 + 4)	0.017	0.009	(−0.001, 0.035)	† 0.054	0.023	(0.009, 0.099)	† 0.097	0.041	(0.016, 0.177)	0.157	0.142	(−0.122, 0.436)

CRP, C-reactive protein. * Tabulated data are: The quantile regression coefficients (Est.), standard errors (SE) and 95% confidence intervals (CI). † Indicates a significant value (at α = 0.05).

## Supplementary Materials

The following are available online at http://www.mdpi.com/2072-6643/10/8/993/s1, Table S1: Food Parameters available in different countries, Table S2: Shows the quantile regression coefficients for children’s Dietary Inflammatory Index treated categorically with 3 levels (level 1 is the reference level) adjusted for sex, age, race, and BMI, NHANES, 2005–2012.
