# Supplementary material for: Design, Development and Construct Validation of the Children’s Dietary Inflammatory Index
Source: Nutrients. 2018 Jul 30;10(8):993. doi: 10.3390/nu10080993 (PMC6115957; doi:10.3390/nu10080993)
Supplement: Supplementary file 1 [file nutrients-10-00993-s001.pdf]

Supplementary Table 1: Food Parameters available in different countries

Countries	N	Vit A	Vit B_1	Vit B_2	Vit B_3	Vit B_6	Folic Acid	Vit B_{12}	Vit D	Vit C	Vit E	Beta Carotene	Energy	Carbs	Fiber	T. Fat	Sat Fat	MUFA	PUFA	Cholesterol	Protein	Alcohol	Fe	Mg	Se	Zn
USA	8127	×	×	×	×	×	×	×	×	×	×	×	×				×			×	×	×	×	×	×	×
Australia	400	×	×	×	×		×		×	×	×		×	×	×	×	×	×	×	×	×		×	×		×
Japan	620	×	×	×	×	×	×	×	×	×	×		×	×	×	×	×	×		×	×	×	×	×		×
Korea	824	×		×	×					×		×	×	×	×	×	×	×	×		×		×			
South Africa	157		×	×		×	×	×							×	×		×	×	×	×		×	×	×	×
Venezuela	92												×	×		×					×					
Spain	125	×	×	×	×	×	×	×	×	×	×		×	×	×											
Belgium	99	×	×	×	×	×	×	×	×	×	×		×	×	×											
Greece	172	×	×	×	×	×	×	×	×	×	×		×	×	×											
Germany	140	×	×	×	×	×	×	×	×	×	×		×	×	×											
France	107	×	×	×	×	×	×	×	×	×	×		×	×	×											
Italy	73	×	×	×	×	×	×	×	×	×	×		×	×	×											
Sweden	102	×	×	×	×	×	×	×	×	×	×		×	×	×											
Austria	128	×	×	×	×	×	×	×	×	×	×		×	×	×											
United Arab Emirates	358	×	×	×	×	×	×	×	×	×	×				×					×			×	×		×
Chile	1543												×	×	×	×	×	×	×	×	×					

Supplementary Table 2: Shows the quantile regression coefficients for children's Dietary Inflammatory Index treated categorically with 3 levels (level 1 is the reference level) adjusted for sex, age, race, and BMI, NHANES, 2005–2012.

	Weighted CRP Levels											
	25th percentile			50th percentile			75th percentile			90th percentile		
	Est.	SE	95% CI	Est.	SE	95% CI	Est.	SE	95% CI	Est.	SE	95% CI
**CDII Level**												
**Quartile 2**	**0.018**	**0.011**	**[-0.003, 0.039]**	**†0.060**	**0.022**	**[0.018, 0.102]**	**†0.109**	**0.050**	**[0.011, 0.206]**	**0.323**	**0.323**	**[-0.131, 0.777]**
**Quartile 3 (3 + 4)**	**0.012**	**0.009**	**[-0.006, 0.030]**	**†0.062**	**0.026**	**[0.010, 0.113]**	**†0.123**	**0.034**	**[0.057, 0.189]**	**†0.25**	**0.115**	**[0.025, 0.475]**

The quantile regression coefficients (Est.), standard errors (SE) and 95% confidence intervals (CI). † Indicates a significant value (at α = 0.05).
